# Characterization of the complete chloroplast genome sequence of *Pistia stratiotes* (Araceae) and its phylogenetic implications

**DOI:** 10.1080/23802359.2020.1768935

**Published:** 2020-05-27

**Authors:** Guoming Quan, Lushu Chen

**Affiliations:** Department of Urban Construction Engineering, Guangzhou City Polytechnic, Guangzhou, P. R. China

**Keywords:** *Pistia stratiotes*, chloroplast genome, phylogenetic analysis, invasive weed

## Abstract

*Pistia stratiotes* is an invasive aquatic weed in South China. In this study, the first complete chloroplast (cp) genome of *P. stratiotes* was reported and phylogenetic analysis was conducted with Araceae species based on the cp genome sequences. The genome is a circular molecule of 164,551 bp in length with 36.00% average GC content and includes a large single-copy region (90,705 bp), a small single-copy region (21,886 bp), and two inverted repeat regions (25,980 bp). It contains a total of 129 genes, including 84 protein-coding genes, 37 tRNA genes, and 8 rRNA genes. The maximum likelihood tree indicated that *P. stratiotes* is related to the genus of *Alocasia.* The cp genome will provide useful molecular data for further phylogenetic and evolutionary analysis of *P. stratiotes*.

*Pistia stratiotes* L., commonly known as water lettuce, is the only surviving species of *Pistia* genus in Araceae. It is a perennial, free-floating aquatic plant which originated in the Tethys region (Renner and Zhang 2004). It was introduced in China during the 16th century. After naturalization, *P. stratiotes* has become a problematic weed and now it is on the second shortlist of alien invasive species in China and widely distributed in tropical and subtropical countries around the world (Liu et al. 2017). In most natural situations, the species grows rapidly and forms dense mats on surface of water bodies, disrupts native aquatic flora and fauna underneath, threats to biodiversity and impedes fishing, water sport, boat traffic and flood control (Khan et al. 2014; Eid 2017). On the other hand, *P. stratiotes* has been shown to have great potential in phytoremediation due to its ability to accumulate pollutants from wastewater, such as heavy metals (Putra et al. 2015), pestcides (Chattoraj et al. 2014), oils (Yang et al. 2014), pharmaceuticals and personal care products (Lin and Li 2016). To further understand its genetic background, here we assembled and characterized the complete chloroplastt (cp) genome sequence of *P. stratiotes*.

Fresh leaf samples of *P. stratiotes* were collected from Baiyun Lake (23°06′32″N, 113°15′53″E), Guangzhou, Guangdong province of China. The specimen was deposited in the herbarium of Guangzhou City Polytechnic (voucher number: GCP0648). High-quality genomic DNA of *P. stratiotes* was extracted from leaves by TIANGEN plant genomic DNA kit and sequenced by Novaseq platform (Illumina, San Diego, CA, USA). Approximately, 9 Gb of raw data of 150 bp paired-end reads were generated and assembled using GetOrganelle (Jin et al. 2019). Genes and the corresponding coding regions were annotated with Geseq (Tillich et al. 2017). Finally, the validated complete cp genome sequence of *P. stratiotes* was submitted to GenBank with accession number MN885890.

The complete cp genome of *P. stratiotes* is 164,551 bp in length with a typical quadripartite structure consisting of a large single-copy (LSC) region of 90,705 bp, a small single-copy (SSC) region of 21,886 bp, and a pair inverted repeat (IR) regions of 25,980 bp. A total of 129 genes were predicted, including 84 protein-coding genes, 37 tRNA genes, and 8 rRNA genes. The overall GC content of the genome is 36.00%. The GC content of three regions is ranked as 55.12, 15.79, and 13.30% for LSC, IR, and SSC, respectively.

In order to confirm the phylogenetic position of *P. stratiotes*, a maximum likelihood analysis was performed by RAxML (Stamatakis 2014) with 1000 bootstrap replicates (Minh et al. 2013; Chernomor et al. 2016) based on 26 complete cp genomes of Araceae, including the typical species of all genera currently registered in GenBank. As shown in Figure 1, *P. stratiotes* is related to the genus of *Alocasia*, consistent with previous studies (Cusimano et al. 2011). The finding will provide valuable molecular data for further phylogenetic and evolutionary analysis.

**Figure 1.**
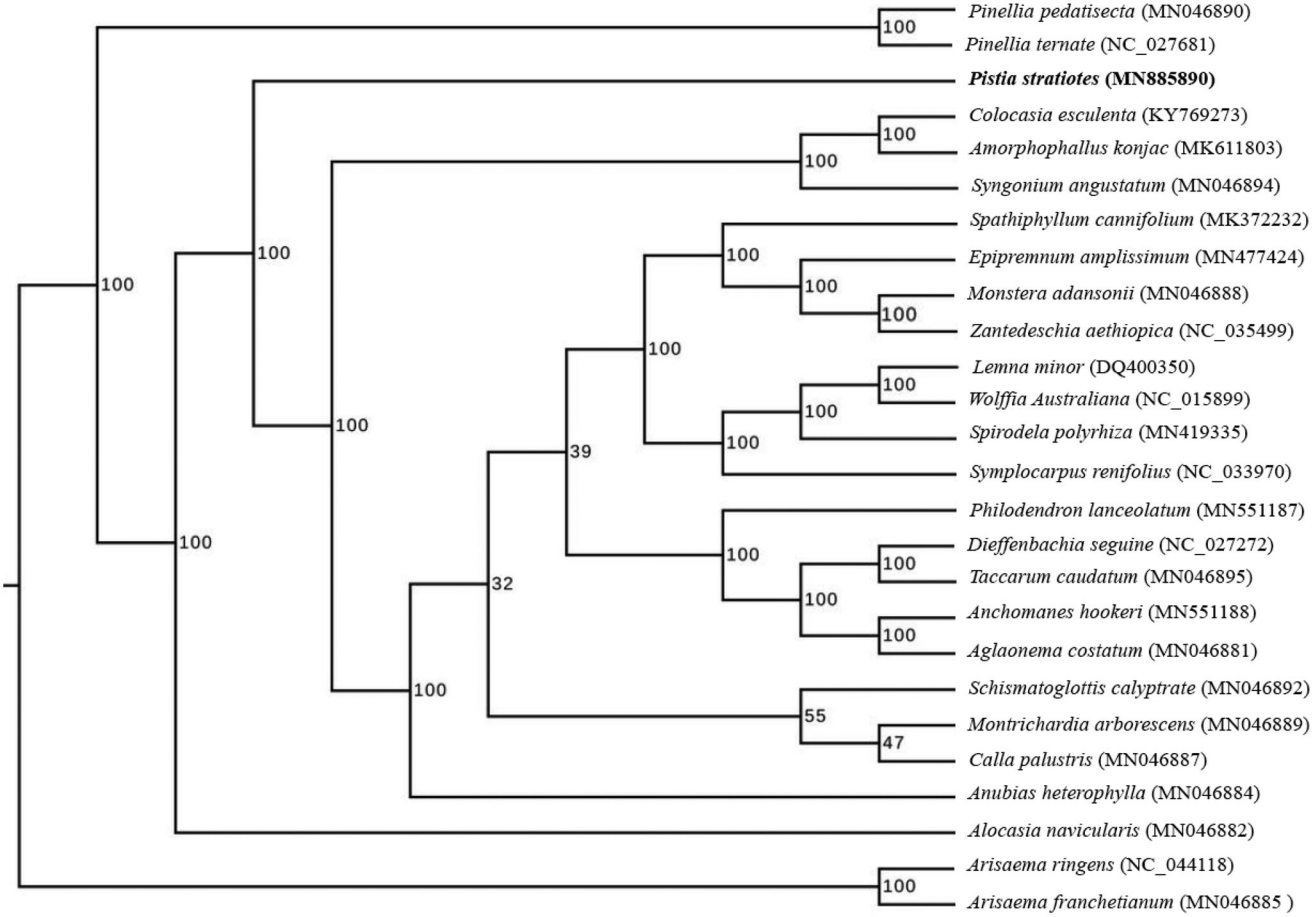
The ML phylogenetic tree based on complete chloroplast genome sequences of 26 species in Araceae. All the sequences were downloaded from NCBI GenBank and the accession numbers were shown in the tree. ML bootstrap support values were indicated at the nodes.

## Data Availability

The data that support the findings of this study are openly available in National Center for Biotechnology Information (NCBI) at https://www.ncbi.nlm.nih.gov/, accession number MN885890.
